# Isotropic Expansion of the Intraprostatic Gross Tumor Volume of Primary Prostate Cancer Patients Defined in MRI—A Correlation Study With Whole Mount Histopathological Information as Reference

**DOI:** 10.3389/fonc.2020.596756

**Published:** 2020-11-23

**Authors:** Maria Kramer, Simon K. B. Spohn, Selina Kiefer, Lara Ceci, August Sigle, Benedict Oerther, Wolfgang Schultze-Seemann, Christian Gratzke, Michael Bock, Fabian Bamberg, Anca L. Grosu, Matthias Benndorf, Constantinos Zamboglou

**Affiliations:** ^1^ Department of Radiation Oncology, Medical Center—University of Freiburg, Faculty of Medicine, University of Freiburg, Freiburg, Germany; ^2^ Institute of Surgical Pathology, Medical Center—University of Freiburg, Faculty of Medicine, University of Freiburg, Freiburg, Germany; ^3^ Department of Urology, Medical Center—University of Freiburg, Faculty of Medicine, University of Freiburg, Freiburg, Germany; ^4^ Department of Radiology, Medical Center—University of Freiburg, Faculty of Medicine. University of Freiburg, Freiburg, Germany; ^5^ Division of Medical Physics, Department of Radiology, Medical Center—University of Freiburg, Faculty of Medicine, University of Freiburg, Freiburg, Germany; ^6^ German Cancer Consortium (DKTK), Partner Site Freiburg, Freiburg, Germany; ^7^ Berta-Ottenstein-Programme, Faculty of Medicine, University of Freiburg, Freiburg, Germany

**Keywords:** MRI, radiotherapy, focal therapy for prostate, histopathologic comparison, prostate cancer

## Abstract

**Introduction:**

An accurate delineation of the intraprostatic gross tumor volume (GTV) is of importance for focal treatment in patients with primary prostate cancer (PCa). Multiparametric MRI (mpMRI) is the standard of care for lesion detection but has been shown to underestimate GTV. This study investigated how far the GTV has to be expanded in MRI in order to reach concordance with the histopathological reference and whether this strategy is practicable in clinical routine.

**Patients and Methods:**

Twenty-two patients with planned prostatectomy and preceded 3 Tesla mpMRI were prospectively examined. After surgery, PCa contours delineated on histopathological slides (GTV-Histo) were superimposed on MRI using *ex-vivo* imaging as support for co-registration. According to the PI-RADSv2 classification, GTV was manually delineated in MRI (GTV-MRI) by two experts in consensus. For volumetric analysis, we compared GTV-MRI and GTV-Histo. Subsequently, we isotropically enlarged GTV-MRI in 1 mm increments within the prostate and also compared those with GTV-Histo regarding the absolute volumes. For evaluating the spatial accuracy, we considered the coverage ratio of GTV-Histo, the Sørensen–Dice coefficient (DSC), as well as the contact with the urethra.

**Results:**

In 19 of 22 patients MRI underestimated the intraprostatic tumor volume compared to histopathological reference: median GTV-Histo (4.7 cm^3^, IQR: 2.5–18.8) was significantly (p<0.001) lager than median GTV-MRI (2.6 cm^3^, IQR: 1.2–6.9). A median expansion of 1 mm (range: 0–4 mm) adjusted the initial GTV-MRI to at least the volume of GTV-Histo (GTV^exp^-MRI). Original GTV-MRI and expansion with 1, 2, 3, and 4 mm covered in median 39% (IQR: 2%–78%), 62% (10%–91%), 70% (15%–95%), 80% (21–100), 87% (25%–100%) of GTV-Histo, respectively. Best DSC (median: 0.54) between GTV-Histo and GTV-MRI was achieved by median expansion of 2 mm. The urethra was covered by initial GTVs-MRI in eight patients (36%). After applying an expansion with 2 mm the urethra was covered in one more patient by GTV-MRI.

**Conclusion:**

Using histopathology as reference, we demonstrated that MRI underestimates intraprostatic tumor volume. A 2 mm–expansion may improve accurate GTV-delineation while respecting the balance between histological tumor coverage and overtreatment.

## Introduction

Depending on risk groups, there is a 10%–30% probability of biochemical recurrence in patients with localized prostate cancer (PCa) after primary whole gland radiation therapy (RT) (1).

An increase of the radiation dose has been proven to be beneficial with regard to a biochemical recurrent free survival after primary PCa RT (2–4). However, the associated increased toxicity to the neighboring organs prohibits a dose-boosting to the entire prostate (5). It has been shown that local recurrence after RT often occurs at the site of the original dominant intraprostatic tumor lesion (6, 7). Consequently, the concept of focal RT in terms of precise dose boosting to the tumor lesion evolved in recent years (8, 9). Involving a high degree of individualization in treatment, focal therapy approaches are an important subject of current research (10). The accurate detection and delineation of the intraprostatic tumor mass are indispensable for successful focal therapy. Multiparametric magnetic resonance imaging (mpMRI) is the standard of care for primary PCa imaging (11, 12) but provides room for improvement regarding tumor lesion detection and volume estimation. Johnson et al. revealed that mpMRI misses 55% of all tumor lesions of which the majority (61%) were smaller than 1 cm (13). A recent study comparing imaging modalities and histopathology showed that MRI underestimates the tumor mass by about half (14). A similar conclusion of underestimation was drawn by Priester et al.: the underestimated GTV-MRI was a third of the real PCa volume in their study (15). Thus, there is evidence that the entire intraprostatic GTV cannot be fully delineated in conventional MRI. Consequently, tumor tissue might partly remain unaffected by focal treatment approaches.

This leads to following questions of this work: Firstly, is it useful to tackle this problem of underestimation by isotropic expansion of the GTV-MRI? Secondly, how far has the GTV-MRI to be expanded to reach a volumetric and spatial accordance with the actual tumor volume and thus to guarantee optimal use of the possibilities of focal therapy based on mpMRI? To answer these questions a thorough comparison between GTV-MRI and a standard of reference is warranted. In this work we chose the intraprostatic tumor volume in co-registered whole-mount pathology sections as the standard of reference.

## Materials and Methods

### Study Design and Patients

For this prospective study we recruited 26 patients with biopsy-proven primary adenocarcinoma of the prostate and subsequent prostatectomy. Each patient preoperatively underwent dedicated prostate MRI. A neoadjuvant androgen deprivation therapy and a previous transurethral resection of the prostate were exclusion criteria in our protocol. The time between MRI and prostatectomy amounted to 36 days on average (range 2–124 days). Mean age and mean PSA were 66 years and 34.9 ng/ml, respectively. We provide further risk criteria according to D´Amico in **Table 1** (16). From all patients written informed consent was obtained. This study was approved by the local ethical review committee (469/14 and 476/19).

**Table 1:** Patients characteristics.

Patient	Age (years)	PSA (ng/ml)	TNM	Gleason score	PCa (% of prostate tissue)
1	52	51.1	pT3b pN1 cM0	5+4 (9)	43%
2	51	17.4	pT3a pN0 cM0	4+3 (7b)	8%
3	59	9.2	pT2c pN0 cM0	4+3 (7b)	5%
4	74	15.0	pT2c pN0 cM0	3+4 (7a)	3%
5	76	20.7	pT2c pN0 cM0	4+3 (7b)	15%
6	59	15.8	pT3b pN1 cM0	4+5 (9)	21%
7	73	40.0	pT3a pN1 cM0	4+5 (9)	21%
8	53	16.3	pT3a pN0 cM0	4+4 (8)	8%
9	72	28.9	pT3b pN1 cM0	4+4 (8)	15%
10	67	218.0	pT3b pN0 cM0	4+4 (8)	55%
11	67	6.1	pT3a pN0 cM0	3+4 (7a)	15%
12	48	23.0	pT3b pN1 cM0	4+3 (7b)	42%
13	66	17.2	pT3 pN0 cM0	4+3 (7b)	16%
14	70	61.0	pT3b pN0 cM0	4+3 (7b)	4%
15	69	103.0	pT3a pN0 cM0	4+5 (9)	7%
16	76	5.0	pT2c pN0 cM0	4+3 (7b)	9%
17	75	17.8	pT2c pN0 cM0	3+4 (7a)	6%
18	53	72.0	pT3b pN1 cM0	5+4 (9)	28%
19	64	19.5	pT3a pN0 cM0	4+4 (8)	7%
20	72	24.8	pT2a cM0 cM0	4+4 (8)	1%
21	74	13.9	pT3a pN0 cM0	4+3 (7b)	7%
22	66	17.5	pT3b pN1 cM1	4+5 (9)	43%
Min	48	5,0		3+4 (7a)	1%
Max	76	218		5+4 (9)	55%
Mean	65	37.0			17%

PSA and cM were defined preoperatively; pT, pN and Gleason score were assessed postoperatively after histopathological diagnostic of the resected prostate and lymph nodes. The sixth column informs about the PCa´s percentage of the prostate tissue with PCa defined using GTV-Histo.

### MRI—Imaging and Contouring

Twenty-two patients (85%) underwent MRI in a 3 Tesla system (Trio Tim, Siemens, Germany: 17 patients; Magnetom Vida, Siemens, Germany: three patients; Skyra, Siemens, Germany: two patients). A 1.5 Tesla system was used in four patients (Aera, Siemens, Germany: three patients; Avanto, Siemens, Germany: one patient). These four patients were retrospectively excluded from our analysis to prevent bias introduced by different MRI standards.

In all patients, axial diffusion weighted images (DWI) and axial T2-weighted turbo spin echo (T2W-TSE) images were acquired and employed for contouring of the prostate and the suspicious tumor areas (MRI-GTV). More detailed information about MR image data can be found in Zamboglou et al. (17). According to a standardized and established reporting system (PI-RADSv2, Prostate Imaging: Reporting and Data System) contours were generated by a board-certified radiologist experienced in the interpretation of prostate MRI (18). The contours were reevaluated by an experienced radiation oncologist and a final consensus volume was created. Both readers were blinded to the histopathological information. For our analysis, we included all areas that were assigned PI-RADS ≥ 3.

### Histopathology

**Figure 1:**
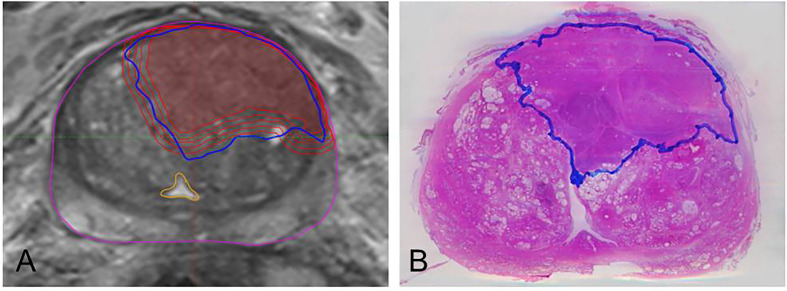
Example of MRI/histopathology registration and isotropic expansion of GTV-MRI. The left image **(A)** depicts step by step expansion of GTV-MRI (red). It illustrates both the underestimated volume (innermost line) compared to GTV-Histo and the nearly complete coverage of those by applying isotropic expansion of 4mm. The yellow structure boarders the urethra which is not targeted by any GTV-MRI or GTV-Histo. Image **(B)** shows a H&E stained slide having been prepared after prostatectomy and containing the GTV-Histo contour (blue).

The resected prostate was marked with ink and fixed for 24 h in formalin. Thereafter we placed the specimen with 6.5% agarose gel in a localizer customized by our Medical Physics Laboratory in order to avoid a change in position in the following steps: We prepared *ex-vivo* computer tomography (CT) scans of every layer previously tagged on the localizer. With a cutting device being also customized in-house, the prostate was sliced every 4 mm on the same level and at the same angle as the *ex-vivo* images were taken. To obtain a histopathological reference, the whole-mount sections were processed to slides stained with Hematoxylin and Eosin (H&E) on which PCa tissue was delineated (**Figure 1**) by one experienced pathologist under microscopic control.

### Registration

The co-registration between images and histopathology was performed according to the already published protocol from our group (17). The slices including the PCa contours were digitized, aligned in the same positions (MITK Workbench 2013.09; German Cancer Research Center) and merged to the associated *ex-vivo* CT applying a manual non-rigid deformation tool in MITK (MITK workbench 2015.5, MITK workbench 2014.10). After matching, the PCa contours were transferred and automatically interpolated to generate GTV-Histo. To guarantee a correct registration of the slices, we made use of the form of the prostate and the urethra which we pierced with a radiopaque drainage before *ex-vivo* imaging. The *ex-vivo* scans including histopathological information were manually co-registered with *in-vivo* MRT (T2w) by using non-rigid deformations. Subsequently, we imported the image data sets into Eclipse ™ (Treatment Planning System, Varian, USA) for further analyses.

### Expansion

In Eclipse™ we enlarged the GTV-MRI in 1 mm steps until an isotropic expansion by 4mm was achieved in every patient (**Figure 1**). If the contour of GTV-MRI overlapped the prostate contour, we cut the outside protruding part in order to perform a prostate constrained expansion. Subsequently, we compared every enlarged volume with GTV-Histo to detect the expansion value yielding a volume at least equal to the histological one (hereafter named GTV^exp^-MRI). To determine the coverage ratio of GTV-Histo by GTV-MRI/GTV^exp^-MRI (tumor coverage) we created intersection-volumes. Since the sole calculation of intersection volumes does not account for a possible overestimation of GTV-Histo, we additionally applied the Dice–Sørensen coefficient (DSC) as an established method to assess agreement between segmentations (19). DSC will be 1 if GTV^exp^-MRI and GTV-Histo are identical and 0 if both volumes are spatially separated. Furthermore, we calculated the percentage of tumor lesions relative to the volume of the whole prostate. In view of the feasibility of focal therapy approaches, we assessed the position of GTV to the urethra by defining whether it is touched or not by GTV-MRI (initial and expanded).

### Statistical Analysis

Microsoft Excel 2016 and GraphPad PRISM v7.04 (GraphPad Software) were used for statistical analysis. D´Agostino-Pearson normality test was applied to test Gaussian distribution. When data were normally distributed, Wilcoxon matched-pairs signed rank test with a threshold for statistical significance p < 0.05 was performed. For data showing normal distribution we tested statistical significance (p < 0.05) with two tailed paired t test. Medians and interquartile ranges (IQR) were calculated.

## Results

### Volumetric Analysis

**Figure 2:**
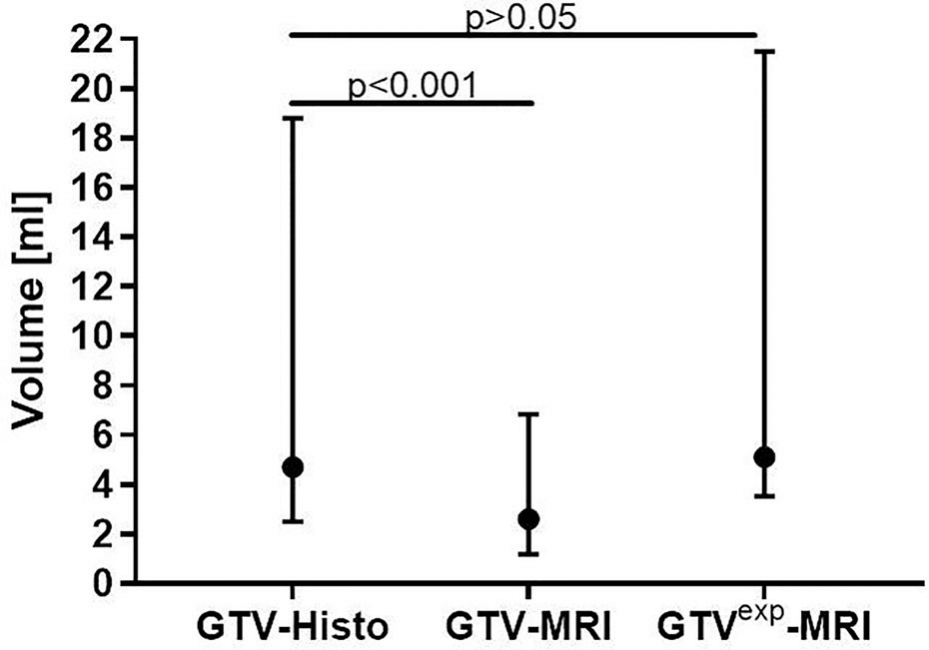
Comparison between absolute volumes of GTV-MRI, GTV-Histo and GTV^exp^-MRI. GTV-MRI (2.6 ml in median) was significantly smaller than GTV-Histo (4.7 ml in median). By applying a 1mm expansion step, GTV^exp^-MRI (median 5.1 ml) had no significant differences to GTV-Histo. Median values with interquartile ranges are shown.

Median GTV-Histo (4.7 ml, IQR: 2.5–18.8 ml) was significantly (p<0.001) larger than median GTV-MRI (2.6 ml, IQR: 1.2–6.9 ml) (**Figure 2**). In 19 of 22 analyzed patients MRI underestimated the intraprostatic tumor volume compared to histopathological reference, three patients showed an inverse relation.

The initial GTV-MRI had to be isotropically enlarged by 1mm in median (range: 0–4 mm) to equal the respective histological volume. The median expanded GTV-MRI was 5.1 ml (IQR: 3.5–21.5 ml).

### Spatial Analysis

In **Table 2** the respective results for each study endpoint after expansion of GTV-MRI with 0–4 mm are presented. The proportion of the covered area of GTV-Histo by GTV-MRI (tumor coverage) for the gradual expansion from 0 to 4 mm was 39% (IQR: 16%–55%), 62% (IQR: 32%–71%), 70% (IQR: 47%–81%), 80% (55–89), 87% (61%–94%), respectively.

**Table 2:** Overview of investigated endpoints after GTV expansion with 0–4 mm.

	Volume [ml]	DSC with GTV-Histo	Coverage of GTV-Histo (%)	% of prostate volume	Overlap with urethra (% of 22 patients)
GTV-Histo	4.7	1	100	12	50
GTV^+0mm^-MRI	2.6	0.46	39	7	36
GTV^+1mm^-MRI	5	0.51	62	12	41
GTV^+2mm^-MRI	6.4	0.52	70	16	41
GTV^+3mm^-MRI	8	0.46	80	21	64
GTV^+4mm^-MRI	9.7	0.44	87%	25%	73%

Column 2 shows results of volumetric analysis and columns 3–6 for spatial assessment. All parameters were defined for GTV-Histo as reference, for the initial delineated GTV-MRI (GTV^+0mm^-MRI) and for every enlarged volume (GTV^+1-4mm^-MRI). Median values over all patients are presented.

**Figure 3:**
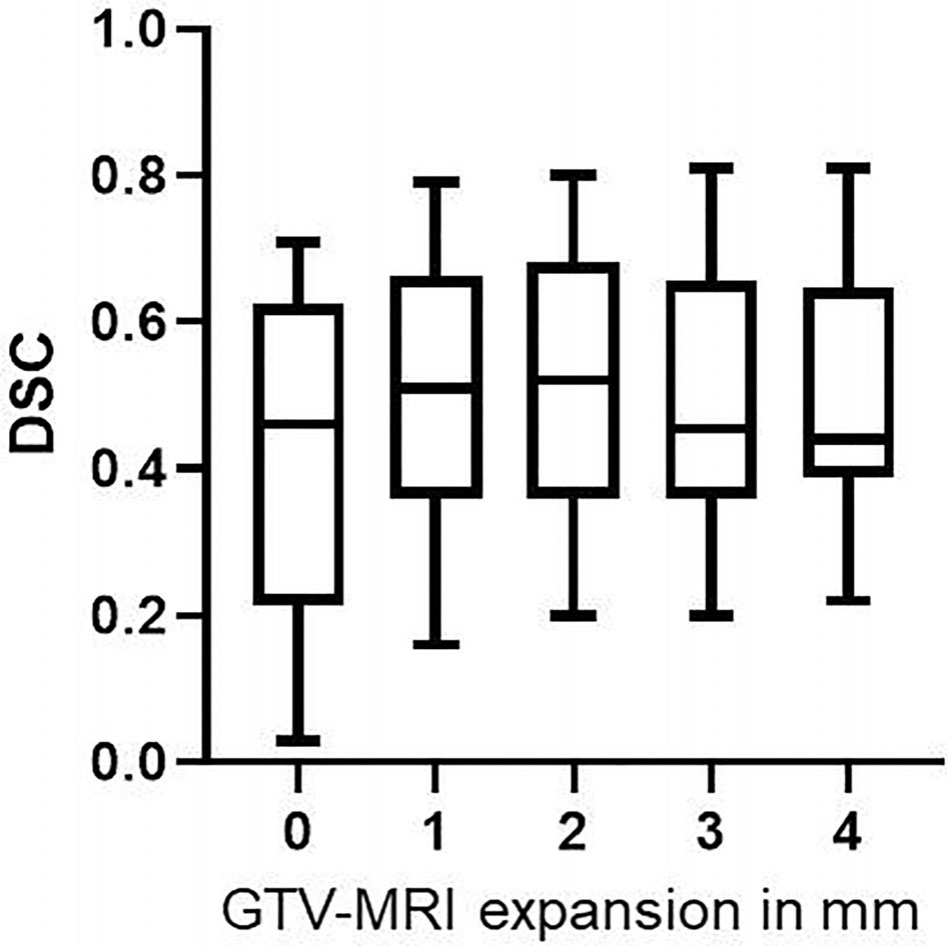
DSC values for GTV-MRI expansion with 0–4 mm. By expanding GTV-MRI with 0–4 mm the median DSC was 0.46 (IQR: 0.22–0.63), 0.51 (IQR: 0.36–0.66), 0.52 (IQR: 0.36–0.68), 0.46 (IQR: 0.36–0.66) and 0.44 (IQR: 0.39–0.65), respectively. Box plots are presented. Expansion with 1 and 2 mm, respectively, led to statistically significant higher DSC (p<0.001 for both) compared non-expanded GTV-MRI.

After expansion of GTV-MRI with up to 4mm the best DSC achieved for all patients was 0.54 which was statistically significant (p<0.001) higher than DSC before expansion (median: 0.46). In median an expansion of 2 mm was required to obtain the best DSC for each individual patient (**Table 3**). In direct comparison between all expansion steps an expansion with 2 mm had the highest DSC with 0.52 (IQR: 0.36–0.68) which was statistically significant (p=0.0002) higher than DSC before expansion (**Figure 3** and **Table 2**).

**Table 3:** DSC values between GTV-Histo and GTV-MRI before and after expansion.

Patient	DSC before expansion	Best DSC after expansion from 0–4 mm	Expansion needed to reach best DSC (mm)
1	0.71	0.71	0
2	0.7	0.7	0
3	0.48	0.69	1
4	0.16	0.38	2
5	0.2	0.4	4
6	0.41	0.51	2
7	0.56	0.73	2
8	0.53	0.53	0
9	0.17	0.4	4
10	0.55	0.75	4
11	0.03	0.22	4
12	0.22	0.41	4
13	0.20	0.35	4
14	0.27	0.61	3
15	0.67	0.78	1
16	0.25	0.43	1
17	0.46	0.55	1
18	0.67	0.76	4
19	0.61	0.61	0
20	0.25	0.42	3
21	0.46	0.51	1
22	0.69	0.81	4
Median	0.46	0.54	2

DSC: Second column shows calculated DSC in median before expansion. The best DSC that was achieved within an expansion frame of 4mm is listed in column 3, the respective expansion reaching this value in column 4.

The median prostate volume was 50.3 ml (IQR: 34.5–63.7 ml). By expanding GTV-MRI with 0–4 mm in median 7% (IQR: 4–16%), 12% (IQR: 9–23%), 16% (IQR: 11–28%), 21% (IQR: 12–33%), and 25% (IQR: 14–39%) of the prostatic volume were covered, respectively. The urethra was covered by initial GTV-MRI in eight patients (36%). Of the 14 patients without initial, an expansion with 1–4 mm yielded to GTVs-MRI overlapping the urethra in additional one (7%), one (7%), six (43%), and eight (57%) cases, respectively.

## Discussion

As focal therapies are increasingly on the rise as part of treatment of primary PCa (20), precise imaging is gaining even more importance. In addition to sensitive detection of tumor lesions a complete coverage of the intraprostatic tumor burden is a crucial prerequisite for successful treatment outcome. At present, mpMRI is the gold standard both for tumor detection and for GTV delineation in focal therapy planning (10, 21). Our study investigated the performance of mpMRI with focus on the tumor volume estimation and coverage based on whole-mount histopathology as reference standard. In the setting of focal therapy planning, we additionally addressed whether and how isotropic expansion of manually delineated PCa lesions in MRI can improve the therapeutic ratio in terms of tumor coverage vs. overtreatment.

Several studies revealed that mpMRI underestimates the size of GTV (14, 15, 22, 23). As a histopathological comparison showed that [^68^Ga]PSMA-PET does not overestimate the intraprostatic tumor volume (19), different definitions of true GTV can be applied: GTV-MRI was compared to whole-mount histopathology by Bettermann et al. and Priester et al. whereas the volume in [^68^Ga]PSMA-PET served as reference in the studies of Zamboglou et al. and Spohn et al. Priester et al. found GTV-MRI to be around one third of the actual tumor volume, whereas the other three studies reported a GTV-MRI of around half the actual tumor volume. The latter results are consistent with the present study. The histological volume (median: 4.7 ml) differed significantly from the GTV delineated in MRI (median: 2.6 ml).

We therefore examined whether expansion of GTV-MRI provides more complete coverage of the whole tumor mass. Our gradually isotropic 1 mm expansion of GTV-MRI firstly aimed at volumetric concordance with GTV-Histo. We discovered an expansion of 1mm as median value was sufficient to adjust the GTV-MRI to the histological volume. This extended GTV was 5.1ml in median (IQR: 3.5–21.5 ml) and thus roughly equal to GTV-Histo.

Supplementary to the volumetric analysis we evaluated the spatial proportion using the DSC and percentage of coverage of GTV-Histo as endpoints. A sparse coverage of GTV-Histo (39%) before expansion increased millimeter by millimeter to 62%, 70%, 80%, and 87% for GTV^+1-4mm^-MRI, respectively. The similar tendency of this finding has been shown by two studies addressing 95% histological tumor coverage based on MRI. Anwar et al. reported a required expansion of 5 mm, Gibson et al. even stated a necessary expansion of 8–9 mm (24, 25). Due to different coverage targets (95% in both other studies vs. at least 70% in our study) the higher values for expansion are not directly comparable to ours. Besides, in both studies MRI was performed with endorectal coil deforming prostate shape and GTV-MRI was not delineated according to the current standardized imaging criteria PI-RADSv2 (26).

Within an expansion until 4 mm, the best DSC has been attained by expanding the GTV-MRI with 2 mm (median). The median DSC resulting from this expansion strategy was 0.54. Accordingly, an expansion with 2 mm led to the highest DSC value (median 0.52) of all expansion steps (0–4 mm), although only a decent difference was observed between 1mm and 2mm expansion. When applying an expansion with 3 and 4 mm, the DSC values decreased. The moderate DSC values are most likely consequence of inaccuracy in volume estimation based on MRI but probably also related to possible inaccuracies in co-registration between MRI and histopathology. Previous histopathological comparison studies stated similar results regarding the spatial overlap between mpMRI and histopathology. An average DSC of 0.48 and 0.37 was reported by Zamboglou et al. and Chang et al., respectively (17, 27). Steenbergen et al. likewise reported moderate agreement between tumor delineation based on MRI and histopathology by calculating a mean kappa index of 0.45 which is identical to DSC when analyzing on voxel-level (28). The inverse relationship between DSC and coverage of GTV-Histo from an expansion of 2mm and larger suggests that with a wider expansion GTV-MRI covers more healthy tissue than tumor tissue. Considering the transfer to the clinical practice, we defined the GTV-MRI´s portion of the total prostate volume ranging from 7%–25% for 0–4 mm expansion, respectively. When applying focal therapy approaches like RT dose escalation, particular efforts should be made to protect the urethra in order to avoid increasing genito-urethral toxicity. Our analysis showed that in 57% of the patients the urethra was affected by 4mm expanded GTV-MRIs when there was no contact before expansion. This clinically relevant fact indicates an appropriately chosen range for expansion in our study. However, the awareness that a complete coverage of histological GTV is partly achieved at the expense of healthy tissue should promote the importance of careful application of GTV-expansion. Fourteen patients did not have contact between GTV-MRI and the urethra before expansion. Of these an expansion with 4 mm leaded to contact with the urethra in eight cases (57%) whereas an expansion with 2 mm decreased the number to one case (7%). Furthermore, an expansion of GTV-MRI by 2 mm encompassed in median 16% of total prostate volume, which should be a feasible target for focal therapies. 

Taking all the mentioned parameters into account (compare for **Table 3**) we propose a 2 mm expansion in order to improve tumor coverage by MRI and to avoid overtreatment at the same time. At 2 mm expansion, we found the highest DSC with GTV-Histo, an only small increase in urethra overlap (one additional patient) and a 70% coverage of tumor tissue by the expanded MRI volume.

Our study is limited by possible inaccuracy in the co-registration process of histopathology and MRI. During the time between prostatectomy and cutting the histological slices, the prostate was prone to shrink discretely in a non-linear way. Additionally, the images taken before and after resection differed in position regarding the angle of the axial slices. In order to prevent a mismatch, we applied a non-rigid registration tool in MITK allowing to rotate and stretch the prostate in all three usual axes. Thereby we made use of the prostate shape, of intrinsic landmarks such as calcifications and of fiducial markers which we positioned into the urethra before the prostate was imaged *ex-vivo*. Although GTV-Histo was considered as the standard of reference, it should be mentioned that Gibson et al. proved that pathologists tend to underestimate the true extent of disease (24).

Another limitation is our cohort of patients being classified as intermediate and high-risk group of PCa. This is due to the fact that the results of our study bases on histopathological reference and we were consequently depended on whole-mount prostate specimen. These patients are not suitable for urological focal therapies such as high intensity focused ultrasound (HIFU), cryotherapy or focal laser ablation (FAL) but may highly benefit from focal RT *via* dose boosting to the tumor lesion (8). Due to the implementation of an elaborate MR/histopathology registration protocol our study included a relatively small number of patients (n=22). We prospectively enlarge our existing database and plan a confirmatory study in the future. Additionally, our results should be validated in future external studies.

Furthermore, our study is only slightly accounting for possible interobserver heterogeneity by using consensus contours of two experienced readers. As the interpretation of prostate MRI has been proven to be complex due to a variety of pitfalls (29), further investigations with various teams of observers are warranted to validate our findings.

## Conclusion

In our histopathological comparison-study we showed that MRI underestimates GTV which can be adjusted to the histological tumor volume by an isotropic 1 mm— expansion. Only modifying the GTV-MRI volume in this manner is not sufficient to achieve total coverage of the histological tumor, though. To accomplish this goal, further expansion is required. An expansion of 2 mm could be employed to balance histological coverage and overtreatment. This strategy may provide more accurate delineation of intraprostatic GTV in MRI and therefore support individuality in treatment of primary PCa in terms of focal therapies.

## Data Availability Statement

The original contributions presented in the study are included in the article/supplementary material. Further inquiries can be directed to the corresponding author.

## Ethics Statement

The studies involving human participants were reviewed and approved by Ethics committee—Medical Center, University of Freiburg. The patients/participants provided their written informed consent to participate in this study.

## Author Contributions

AG, MBe, FB, and CZ did the conceptual work. MBe and CZ delineated the contours. CG and WS-S performed the surgeries. AS and MK recruited the patients. MBo, FB, BO, and MBe defined the MRI protocols. SK performed histopathology work-up. MK, SS, and LC did the MRI/histology fusion. MK and CZ performed the data analyses. MK, CZ, and MBe drafted the manuscript. All authors contributed to the article and approved the submitted version.

## Funding

CZ received grants from the Klaus Tschira Stiftung (00.014.2019).

## Conflict of Interest

The authors declare that the research was conducted in the absence of any commercial or financial relationships that could be construed as a potential conflict of interest.
